# In Vitro and Ex Vivo Evaluation of a Multi-Target Combination of Plant Extracts and Policosanols: Effects in Mitigating Heart Inflammation and Oxidative Stress

**DOI:** 10.3390/foods15091500

**Published:** 2026-04-25

**Authors:** Lucia Recinella, Giorgia Bray, Angelica Pia Centulio, Davide Ciaramellano, Annalisa Chiavaroli, Gianluca Genovesi, Giustino Orlando, Alessandra Acquaviva, Valentina Citi, Serena Veschi, Anna Piro, Alessandro Cama, Alma Martelli, Vincenzo Calderone, Luigi Brunetti, Sheila Leone

**Affiliations:** 1 Department of Pharmacy, G. d’Annunzio University of Chieti-Pescara, 66013 Chieti, Italy; lucia.recinella@unich.it (L.R.); angelicapia.centulio@phd.unich.it (A.P.C.); d.ciaramellano@unidav.it (D.C.); annalisa.chiavaroli@unich.it (A.C.); gianluca.genovesi@phd.unich.it (G.G.); giustino.orlando@unich.it (G.O.); alessandra.acquaviva@unich.it (A.A.); veschi@unich.it (S.V.); anna.piro@unich.it (A.P.); alessandro.cama@unich.it (A.C.); sheila.leone@unich.it (S.L.); 2 Department of Pharmacy, University of Pisa, 56126 Pisa, Italy; giorgia.bray@phd.unipi.it (G.B.); valentina.citi@unipi.it (V.C.); alma.martelli@unipi.it (A.M.); vincenzo.calderone@unipi.it (V.C.); 3 Department of Human Sciences, Law, and Economics, Telematic University “Leonardo Da Vinci”, UNIDAV, 66100 Torrevecchia Teatina, Italy; 4 Interdepartmental Research Center “Nutrafood: Nutraceutica e Alimentazione per la Salute”, University of Pisa, 56126 Pisa, Italy; 5 CISUP (Centre for Instrumentation Sharing of Pisa University), 56126 Pisa, Italy

**Keywords:** combination of plant extracts, heart, inflammation, oxidative stress, functional foods, nutraceuticals, bioactive compounds

## Abstract

Heart inflammation and oxidative stress are pivotal pathological drivers in the pathophysiology of various cardiovascular diseases. The present study aims to investigate the beneficial effects induced by extracts derived from edible plants, such as *Olea europaea*, and sugar cane on heart health. In particular, we investigated the effects of a novel combination constituting *Olea europaea*, *Scutellaria baicalensis*, and policosanol extracts on heart, in in vitro and ex vivo models. *Olea europaea*, *S. baicalensis*, policosanol extracts and their combination prevented H_2_O_2_-induced reduction in H9c2 cell (immortalized myoblasts, isolated from rat heart tissue) viability. Moreover, pre-incubation with the combination significantly reduced H_2_O_2_-induced ROS levels in the same cells. Our present findings also showed that *Olea europaea*, *S. baicalensis* and policosanol extracts, as well as their combination, increased lipopolysaccharide (LPS)-induced catalase gene expression at all concentrations tested, in mouse heart specimens. In addition, we also observed that *Olea europaea*, *S. baicalensis* and policosanol extracts, as well as their combination, significantly inhibited LPS-induced inducible nitric oxide synthase, cyclooxygenase-2, nuclear factor-kB, and tumor necrosis factor-α gene expression, in the same experimental model. Interestingly, the combination was more effective at decreasing the mRNA levels of all pro-inflammatory markers investigated. Finally, the combination was also able to suppress LPS-induced B-type natriuretic peptide and cardiac troponin I gene expression ex vivo. In conclusion, these findings suggest that this plant-based combination could offer potential benefits for cardiovascular health and support overall heart function in humans.

## 1. Introduction

Cardiovascular diseases (CVDs) are recognized as the major cause of mortality in the world. In particular, several studies have shown the key role exerted by inflammation and oxidative stress in the pathophysiology of CVD, including hypertension, acute coronary syndromes, heart failure, and atherosclerosis [1,2,3,4].

Accordingly, an imbalance between pro-inflammatory and anti-inflammatory markers, as well as between pro-oxidants and antioxidants, has also been suggested to be intimately involved in CVD development and progression [5,6]. In addition, different cardiac biomarkers can be used as indicators of CVD [7]. In particular, B-type natriuretic peptide (BNP), cardiac troponin I (cTnI) and cardiac troponin T (cTnT) exert a pivotal role in CVD progression, and could provide meaningful information about CVD prognosis and/or guide clinical decision making. These biomarkers can be modified by various synthetic drugs, such as statins, angiotensin-converting enzyme (ACE) inhibitors, and β and calcium channel-blockers, the critical role of which, in the treatment of CVD, is well known [8].

Recently, there is increasing attention towards the efficacy of herbal treatments, as well as bioactive compounds found in functional foods, regarding their role in chronic disease prevention. While herbal extracts are not a replacement for conventional medical treatment, a growing body of research suggests that some of them can be beneficial in preventing and managing risk factors for cardiac damage. In particular, herbal extracts and their bioactive compounds offer promising cardioprotective effects, enhancing cardiovascular health. Moreover, it is well known that a diet rich in herbal-derived antioxidant compounds, with particular regard to phenolic compounds, is critically involved in cardiovascular health promotion [9]. In particular, phenolic compounds, like those found in *Olea europaea*, are crucial for promoting cardiovascular longevity and mitigating risk factors associated with heart damage [10].

In addition, the combined use of herbal extracts may increase their beneficial effects, by allowing for synergism, acting on a number of pathways simultaneously, and decreasing doses of single components as well as reducing their side effects [11].

*Scutellaria baicalensis* Georgi, also known as Huang-qin or Chinese skullcap, is obtained from the dehydrated root of *Scutellaria baicalensis* (Lamiaceae) [12]. The most prominent components isolated from the *S. baicalensis* roots can be classified into the following: free flavoinoids (including baicalein and wogonin), flavonoid glycosides (such as baicalin and wogonoside), phenylethanoid glycosides, and others. In particular, baicalein, baicalin, and wogonin exert different biological properties, including antioxidant, anti-inflammatory, antiviral, hepatoprotective, and neuroprotective activities [13,14,15,16]. Recently, Zhao and collaborators (2019) reported that *S. baicalensis* has been used in the treatment of several disorders, such as hypertension, inflammation, and tumors, as well as immunologic disorders [12].

Olive (*Olea europaea* L.) leaves contain bioactive components, such as phenols, including flavonoids (luteolin, apigenin, etc.), hydroxytyrosol, tyrosol, ferulic and caffeic acids, and secoiridoids, mainly including oleuropein and dimethyloleuropein. The main polyphenolic compounds of olive leaves include oleuropein, hydroxytyrosol and tyrosol [17]. In particular, a number of studies have reported that olive leaves induce cardioprotective activities, such as anti-inflammatory, antiatherogenic and antihypertensive effects [18,19,20]. Furthermore, polyphenolic constituents in the olive plant, such as oleuropein, flavonoids, hydroxytyrosol, chalcones, and tannins, have been described to possess protective effects on the cardiovascular system [21]. Intriguingly, oleuropein, its most prominent component, displays several protective activities against hypertension [20], atherosclerosis [21], and diabetes [22]. In particular, the presence of polyphenolic compounds (as oleuropein and analogs), which are able to inhibit L-type calcium channels, was found to be involved in the antihypertensive properties of olive leaves, as well as in olive oil [23].

Policosanols are a mixture of high-molecular-weight aliphatic alcohols, derived from purified sugar cane wax (*Saccharum officinarum* L.). This mixture mainly includes docosanol, tetracosanol, hexacosanol, octacosanol, triacontanol, dotriacontanol, and tetratriacontanol, with octacosanol, triacontanol and hexacosanol being the major constituents [24]. In particular, octacosanol has been reported to display anti-inflammatory activity [25]. Policosanol administration was shown to decrease the risk of atheroma formation through reductions in endothelial damage, platelet aggregation, and foam cell formation in rodents [26].

On the basis of this evidence, the present study aims to investigate the beneficial effects exerted by extracts derived from edible plants, such as *Olea europaea*, and sugar cane on heart health. In this context, the preventive effects of *Olea europaea*, *S. baicalensis*, and policosanol extracts, as well as three concentrations of their combination (MIX-1, MIX-2, and MIX-3), were evaluated in an in vitro model of H_2_O_2_-induced oxidative stress, constituting H9c2 cells (immortalized myoblasts, isolated from rat heart tissue).

Moreover, we investigated the cardioprotective effects of the same extracts, both alone and in combination (MIX-1, MIX-2, and MIX-3), on mouse heart specimens treated with *Escherichia coli* lipopolysaccharide (LPS), which represents a validated ex vivo experimental model of tissue inflammation [27,28]. In this context, we measured the gene expression of different biomarkers involved in oxidative stress and inflammation, as well as in cardiac injury, in the same ex vivo experimental model, after treatment with the extracts, alone and in combination.

The final objective of this study is also to identify the best combination to use as a possible plant-based remedy for heart health.

## 2. Materials and Methods

### 2.1. Characteristics of Natural Ingredients

*Olea europaea* (leaf and fruits (total oleuropein content = 15% p/p), *S. baicalensis* root (total baicalin content = 30.2% p/p) and policosanol (total octacosanol content = 62.77%) extracts (Table 1) were supplied as dried powder by Difass International S.p.a. (Coriano, Rimini, Italy). The chemical characterization and standardized content of the natural extracts used in this study were provided by Difass International S.p.a., Italy, based on HPLC, for *Olea europaea* and *S. baicalensis* extracts, and on GC for policosanol extracts analysis. *Olea europaea* and *S. baicalensis* extracts were dissolved in sterile phosphate-buffered solution (PBS). Policosanol extracts were dissolved in 30:70 (ethanol:water) solution. Then, for the experimental evaluations, the stock solution was dissolved in sterile PBS.

### 2.2. Toxicological and Pharmacological Studies

#### 2.2.1. Cell Cultures

H9c2 cells (immortalized rat heart myoblasts, ATCC, Manassas, VA, USA) were cultured in Dulbecco’s modified Eagle Medium with high glucose (DMEM-HG, Sigma-Aldrich, St. Louis, MO, USA), with the addition of 10% Fetal Bovine Serum (FBS), 1% penicillin-streptomycin solution containing 10,000 units of penicillin and 10 mg/mL of streptomycin (Sigma-Aldrich, St. Louis, MO, USA). Cells were incubated in T75 red cap tissue culture flasks, at 37 °C, in a humidified atmosphere with 5% CO_2_. The cells were used at approximately 80% confluency for each experiments.

#### 2.2.2. Evaluation of Effects of *Olea europaea*, *S. baicalensis* and Policosanol Extracts on Cell Viability

H9c2 cells were seeded in a 96-well cell culture plate at a density of 20 × 10^3^ cells per well. After 24 h, the culture medium was replaced, and the cells were treated with *Olea europaea* (5, 50 and 500 µg/mL), *S. baicalensis* (10, 100 and 1000 µg/mL), and policosanols (1.5, 15 and 150 µg/mL). After a further 24 h, cell viability was assessed using a Water-Soluble Tetrazolium-1 (WST-1) colorimetric assay. The detailed protocol is described in the Supplementary Materials section.

#### 2.2.3. Preventive Effects of *Olea europaea*, *S. baicalensis* and Policosanol Extracts, and Their Combination, in an In Vitro Model of H_2_O_2_-Induced Oxidative Stress

H9c2 cells were seeded in a 96-well cell culture plate with a density of 20 × 10^3^ cells per well. After 24 h, the culture medium was replaced, and the cells were treated with the control or with the extracts tested individually, which were as follows: *Olea europaea* (5, 50 and 500 µg/mL), *S. baicalensis* (10, 100 and 1000 µg/mL), and policosanols (1.5, 15 and 150 µg/mL). In addition, MIX-1 [*Olea europaea* (5 µg/mL) + *S. baicalensis* (10 µg/mL) + policosanols (1.5 µg/mL)], MIX-2 [*Olea europaea* (50 µg/mL) + *S. baicalensis* (100 µg/mL) + policosanols (15 µg/mL)] and MIX-3 [*Olea europaea* (500 µg/mL) + *S. baicalensis* (1000 µg/mL) + policosanols (150 µg/mL)] were also tested.

After 24 h, without removing the previous treatments, a freshly prepared hydrogen peroxide solution (H_2_O_2_) in an amount of 200 µM was added for 2 h [29]. At the end of the treatment, the cell viability was evaluated using the cell proliferation probe WST-1 (Roche, Basilea, Switzerland). The detailed protocol is described in the Supplementary Materials section.

### 2.3. Ex Vivo Studies

#### 2.3.1. Animals and Ethical Directives

Adult C57/BL6 male mice (3-month-old, weight 20–25 g) (*n* = 30, 6 for each experimental group) were housed in Plexiglas cages. Housing conditions and experimentation procedures were strictly in agreement with the European Community ethical regulations (EU Directive no. 26/2014) on the care of animals for scientific research. Approval of the study protocol was performed by a local ethical committee (‘G. d’Annunzio’ University, Chieti, Italy) and the Italian Health Ministry (Project no. F4738.N.ZDZ).

#### 2.3.2. Animal Protocol

Isolated specimens of the heart were incubated in a humidified incubator with 5% CO_2_ at 37 °C for 4 h (incubation period), in RPMI buffer with added bacterial LPS (10 µg/mL) [27,28,30]. During the incubation period, the heart tissues were challenged with *Olea europaea* (5, 50 and 500 µg/mL), *S. baicalensis* (10, 100 and 1000 µg/mL), policosanols (1.5, 15 and 150 µg/mL) extracts, MIX-1 [*Olea europaea* (5 µg/mL) + *S. baicalensis* (10 µg/mL) + policosanols (1.5 µg/mL) extracts]; MIX-2 [*Olea europaea* (50 µg/mL) + *S. baicalensis* (100 µg/mL) + policosanols (15 µg/mL) extracts] and MIX-3 [*Olea europaea* (500 µg/mL) + *S. baicalensis* (1000 µg/mL) + policosanols (150 µg/mL) extracts] or a control.

#### 2.3.3. Gene Expression Analysis

Extraction of the total RNA was performed from the heart specimens using TRI Reagent (Sigma–Aldrich, St. Louis, MO, USA), in agreement with the manufacturer’s protocol. The gene expression of glutathione peroxidase (*GPx*), catalase (*CAT*), inducible nitric oxide synthase (*iNOS*), cyclooxygenase (*COX*)-2, nuclear factor-kB (*NF-kB*), tumor necrosis factor- α (*TNF-α*), *cTnT*, *cTnT* and *BNP* was studied by quantitative real-time PCR using TaqMan probe-based chemistry [27,28,30]. The detailed protocol is described in the Supplementary Materials section. Gene expression was relatively quantified by the comparative 2^−∆∆Ct^ method [31].

### 2.4. Antioxidant Effects of Olea europaea, S. baicalensis and Policosanol Extracts, and Their Combination, in an In Vitro Model of H_2_O_2_-Induced Oxidative Stress

H9c2 cells were seeded at 30 × 10^3^ cells/well in 96-well black plate coated with a 1% aqueous solution of gelatin (Sigma-Aldrich, St. Louis, MO, USA). After 24 h, the cells were treated with MIX-1, MIX-2 and MIX-3.

After 24 h, without removing the previous treatments, a freshly prepared H_2_O_2_ solution in an amount of 200 µM was added for 2 h. At the end of the treatment, the culture medium was removed, and reactive oxygen species (ROS) were measured using the fluorescence probe dihydroethidium (DHE) (Sigma-Aldrich, St. Louis, MO, USA). The detailed protocol is described in Supplementary Materials section.

### 2.5. Statistical Analysis

Statistical analyses were performed using GraphPad Prism 8.0.2 (Graphpad Software Inc., San Diego, CA, USA). Data are expressed as the mean ± standard error (SEM). Three different experiments were performed, each with three replicates (*n* = 9). Data were analyzed using one-way analysis of variance (ANOVA), followed by a Bonferroni test. Statistically significant differences were defined as *p* < 0.05.

The number of animals randomized for each experimental group was calculated on the basis of the “Resource Equation” N = (E + T)/T (10 ≤ E ≤ 20) [32].

## 3. Results and Discussion

The highest concentration tested (1000 µg/mL for *S. baicalensis*) was chosen based on the concentration range (1–1000 µg/mL) routinely utilized for plant extracts in our established experimental model [27,28,30]. The selection of relative ratios for *Olea europaea*, *S. baicalensis*, and policosanol extracts in the three combinations [MIX-1 [*Olea europaea* (5 µg/mL) + *S.* (10 µg/mL) + policosanols (1.5 µg/mL)], MIX-2 [*Olea europaea* (50 µg/mL) + *S. baicalensis* (100 µg/mL) + policosanols (15 µg/mL)] and MIX-3 [*Olea europaea* (500 µg/mL) + *S. baicalensis* (1000 µg/mL) + policosanols (150 µg/mL)] was based on the standard daily dosages typically employed for the individual constituents in human nutraceutical supplementation. This approach could underscore the potential for integrating these extracts into functional food formulations or dietary supplements to provide multi-target protection against inflammatory-based cardiovascular disorders. Starting from the maximum dosage (MIX-3), two serial dilutions (1:10 and 1:100, represented by MIX-2 and MIX-1, respectively) were tested to characterize the dose-dependent response.

### 3.1. Evaluation of the Effects Induced by Olea europaea, S. baicalensis and Policosanols on H9c2 Cell Viability

**Figure 1:**
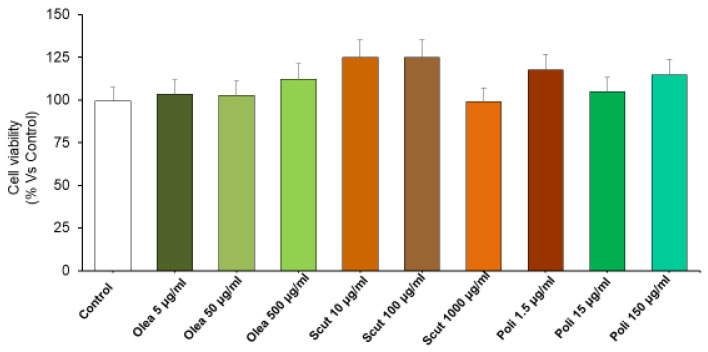
Toxicity of *Olea europaea, S. baicalensis* and policosanol extracts. The graph shows the cell viability (%) of H9c2 treated with *Olea europaea* (5, 50, and 500 µg/mL), *S. baicalensis* (10, 100 and 1000 µg/mL), policosanol (1.5, 15 and 150 µg/mL) extracts. All the experiments were performed in triplicate at minimum (*n* = 9). Data are shown as the mean ± SEM. Statistical significance was calculated by one-way ANOVA followed by a Bonferroni post-test.

H9c2 cells were treated for 24 h with increasing concentrations of *Olea europaea* (5, 50, and 500 µg/mL), *S. baicalensis* (10, 100, and 1000 µg/mL), and policosanol (1.5, 15, and 150 µg/mL) extracts to assess potential cytotoxicity. Our present results (Figure 1), showing that *Olea europaea*, *S. baicalensis*, and policosanol extracts did not modify the viability of H9c2 cells at any concentration, are in agreement with previous studies showing that *S. baicalensis* and *Olea europaea* extracts did not affect the viability of normal cells in vitro [33,34]. The present findings highlight the absence of cytotoxic effects in these experimental conditions and support further investigations about their protective properties against oxidative stress and inflammation.

### 3.2. Evaluation of the Cell Viability Preservation Against H_2_O_2_-Induced Cell Damage in H9c2 Cells

Both inflammation and oxidative stress were reported to be critically involved in a number of CVDs, such as fibrosis, heart failure, left ventricular hypertrophy, diastolic dysfunction, and hypertension, as well as ischemia/reperfusion damage [35].

In the present study, we evaluated the preventive effects induced by *Olea europaea*, *S. baicalensis*, and policosanol extracts alone and in combination (MIX-1, MIX-2, MIX-3) in an in vitro model of H_2_O_2_-induced oxidative stress, in immortalized rat cardiomyoblast H9c2 cells.

**Figure 2:**
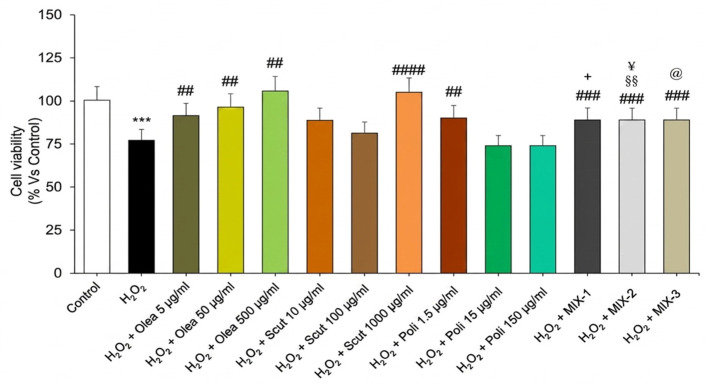
Preventive effect of *Olea europaea, S. baicalensis, and* policosanol extracts, as well as if MIX-1, MIX-2 and MIX-3, against H_2_O_2_-induced cell death. Graphs the show cell viability (%) of H9c2 pre-incubated with *Olea europaea* (5, 50, and 500 µg/mL), *S. baicalensis* (10, 100 and 1000 µg/mL), and policosanol (1.5, 15 and 150 µg/mL) extracts, as well as of MIX-1, MIX-2 and MIX-3, followed by exposure to H_2_O_2_. All the experiments were performed in triplicate at minimum (*n* = 9). Data are displayed as the mean ± SEM. Statistical significance was calculated by one-way ANOVA followed by a Bonferroni post-test. * indicates a significant difference vs. the control (*** *p* < 0.0001); # indicates significant difference vs. H_2_O_2_ (^##^ *p* < 0.01, ^###^ *p* < 0.001, ^####^ *p* < 0.0001); § indicates a significant difference vs. policosanols at 15 µg/mL (§§ *p* < 0.01); @ indicates a significant difference vs. policosanols at 150 µg/mL (@ *p* < 0.05); + indicates a significant difference vs. *S. baicalensis* at 10 µg/mL (+ *p* < 0.05); ¥ indicates a significant difference vs. *S. baicalensis* at 100 µg/mL (*p* < 0.05).

The potential preventive effects of *Olea europaea*, *S. baicalensis*, and policosanol extracts, as well as MIX-1, MIX-2, and MIX-3, against an oxidative stimulus observed after the administration of H_2_O_2_ were tested on H9c2 cells. The treatment with H_2_O_2_ induced a significant decrease in cell viability (Figure 2). This decrease was significantly prevented, in a concentration-dependent manner, by pre-incubation with *Olea europaea* extract, in the entire concentration range evaluated (Figure 2). *S. baicalensis* extract exhibited a significant preventive effect only at the highest concentration tested (Figure 2). In contrast, policosanols showed a significant preventive effect only at the lowest concentration tested, while higher concentrations did not yield significant protection (Figure 2).

Furthermore, pre-incubation with MIX-1, MIX-2 and MIX-3 significantly protected H9c2 cells against oxidative stimuli (Figure 2).

Notably, MIX-1 exhibited greater cytoprotection than *S. baicalensis* did at 10 µg/mL, while MIX-2 exhibited greater cytoprotection than both *S. baicalensis* at 100 µg/mL and policosanols at 15 µg/mL. Furthermore, MIX-3 showed a marked improvement in efficacy compared with policosanols at 150 µg/mL (Figure 2).

These observations suggest a possible additive interaction between the components in the mixtures. Such an additive effect is particularly relevant in oxidative stress-related pathologies, in which multifaceted mechanisms are involved, and single-agent therapies may not provide adequate protection.

### 3.3. Protective Effects of Olea europaea, S. baicalensis, and Policosanol Extracts, as Well as of MIX-1, MIX-2 and MIX-3, on Mouse Heart Specimens Stimulated with LPS

Finally, we investigated the protective effects induced by *Olea europaea*, *S. baicalensis*, and policosanol extracts, and their combinations in three dosages, MIX-1, MIX-2 and MIX-3, in mouse heart specimens treated with LPS, which constitutes a validated experimental model with which to study the activities of plant-derived extracts in modulating inflammatory response and oxidative stress [27,28].

Previous studies have also suggested the role of iNOS as a main contributor in CVDs like arteriosclerosis [36]. Moreover, iNOS activity has been suggested to be associated with endothelial dysfunction, as confirmed by previous studies showing increased iNOS protein expression in mice with LPS-induced endothelial dysfunction [37].

In addition, the protective effects induced by a core antioxidant enzyme, CAT, in contrasting atherosclerosis, endothelial dysfunction, and myocardial damage have been demonstrated in several reports [38,39]. Furthermore, CAT deficiency is also associated with various oxidative stress-related diseases such as CVDs [40,41], suggesting a link between reduced antioxidant defenses, increased oxidative stress, and CVD progression.

The imbalance between antioxidant capacity, including increased CAT activity, and ROS generation, is also well known to contribute to protein, lipid, and DNA oxidative damage, exacerbating CVDs [42].

Interestingly, Schnabeland and collaborators (2005) reported a relationship between GPx-1 activity in whole-blood and CVD development and severity [43]. In addition, GPx-1 levels were suggested to be a useful marker for CV event detection, and acute myocardial infarction incidence was increased in patients with GPx-1 activity impairment [44]. Furthermore, a reduction in GPx levels was found in patients with CVD compared to those of controls, further confirming the crucial role of oxidative stress in CVD onset and progression [45].

In this context, we evaluated the effects of *Olea europaea*, *S. baicalensis*, and policosanol extracts, as well as of MIX-1, MIX-2 and MIX-3, on the gene expression of pro- and anti-oxidant mediators, such as *GPx*, *CAT* and *iNOS*, on isolated LPS-exposed heart specimens, by RT-PCR analysis.

**Figure 3:**
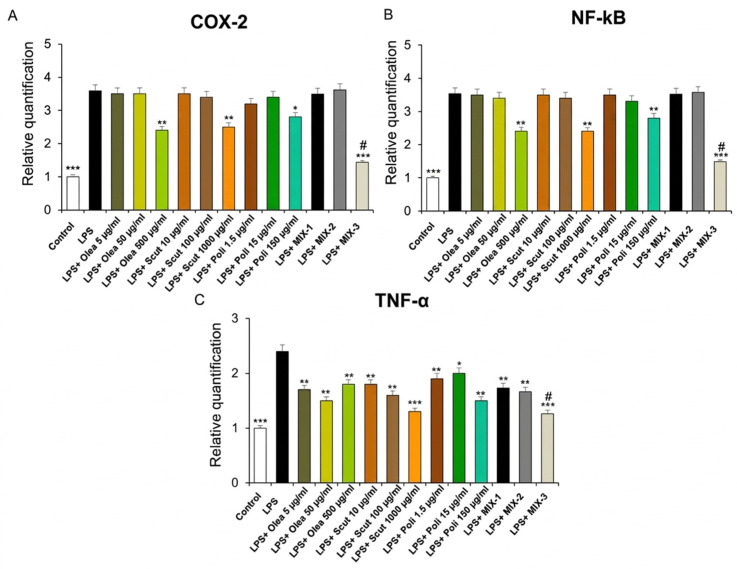
Effects of *Olea europaea*, *S. baicalensis*, and policosanol extracts, as well as of MIX-1, MIX-2 and MIX-3, on LPS-induced glutathione peroxidase (*GPx*) (**A**), catalase (*CAT*) (**B**), and inducible nitric oxide synthase (*iNOS*) (**C**) gene expression (RQ, relative quantification) in mouse heart specimens (*n* = 30, 6 for each experimental group). Data are shown as the mean ± SEM. Statistical significance was calculated by one-way ANOVA followed by a Bonferroni post-test. * *p* < 0.05, ** *p* < 0.005; *** *p* < 0.01 vs. LPS; # *p* < 0.05 vs. LPS + MIX-1 and LPS + MIX-2.

Our present findings showed that *Olea europaea*, *S. baicalensis* and policosanol extracts, as well as their combinations, increased CAT gene expression at all concentrations tested but did not influence GPx mRNA levels. In addition, *Olea europaea*, *S. baicalensis* and policosanol extracts were able to contrast the release of the pro-oxidative marker iNOS, induced by LPS, in the mouse heart, only at a higher concentration (Figure 3). Interestingly, their combination reduced the gene expression of iNOS; however, MIX-3 was more effective at decreasing *CAT* and *iNOS* gene expression compared to MIX-1 and MIX-2 (Figure 3).

In line with this, olive leaf extracts were found to be capable of exerting protective effects in contrasting lipid peroxidation and increasing the levels of antioxidant enzymes, such as CAT, in the human cervical adenocarcinoma HeLa cell line [46]. In particular, the cardioprotective activity of oleuropein, the main secoiridoid from olive tree, is well known. In this context, the beneficial activities of oleuropein could be attributed to its antioxidant capacity, as well as to its modulatory abilities, for several targets, including NO, iNOS, interleukin (IL)-8, and TNFα [47]. The antioxidant effects induced by *S. baicalensis* are also well known. More recently, a significant improvement in antioxidant function, along with a significant decrease in inflammation, including reduced γ-glutamyltransferase and TNF-α, as well as increased CAT serum levels, was found in mice treated with *S. baicalensis* extract [48]. Moreover, the antioxidant activities of policosanols have also been reported and confirmed via decreasing ROS formation as well as increasing CAT activity [49].

Inflammation was suggested to be critically involved in CVD development and progression [50,51]. Notably, pro-inflammatory biomarkers could exert a potential role in predicting the risk for CVD developing and could correlate with CVD severity [52].

Increased blood inflammatory cytokines were reported in heart failure, as previously reported [53,54]. Moreover, NF-kB was shown to be involved in the transcription of different proinflammatory cytokines, including IL-6 and TNF-α [55], which play a key role in mediating cardiac dysfunction [56]. Recently, higher circulating levels of TNF-α and IL-1β were also found in patients with myocarditis [57].

**Figure 4:**
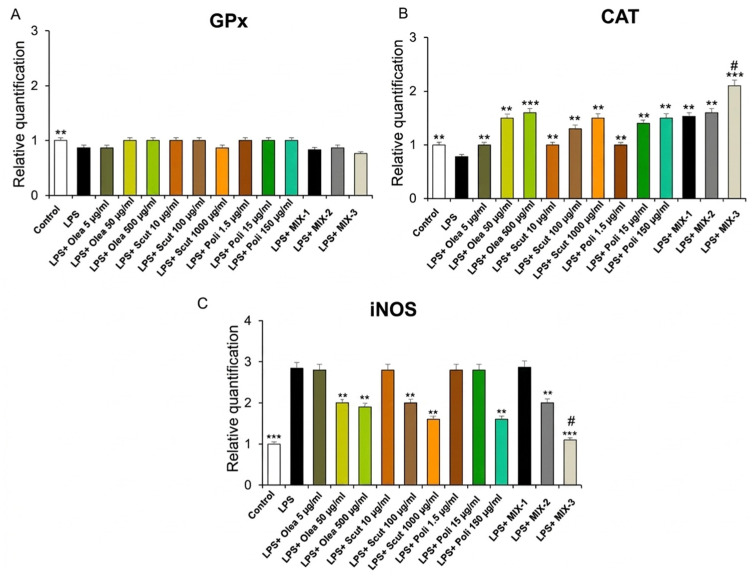
Effects of *Olea europaea*, *S. baicalensis*, and policosanol extracts, as well as of MIX-1, MIX-2 and MIX-3, on LPS-induced cyclooxygenase-2 (*COX-2*) (**A**), nuclear factor kB (*NF-kB*) (**B**), and tumor necrosis factor-α (*TNF-α*) (**C**) gene expression (RQ, relative quantification) in mouse heart specimens (*n* = 30, 6 for each experimental group). Data are shown as the mean ± SEM. Statistical significance was calculated by one-way ANOVA followed by a Bonferroni post-test. ** *p* < 0.005; *** *p* < 0.01 vs. LPS; # *p* < 0.05 vs. LPS + MIX-1 and LPS + MIX-2.

In our ex vivo experimental model, we also reported that *Olea europaea*, *S. baicalensis* and policosanol extracts, at higher concentrations, as well as MIX-3, significantly inhibited *COX-2* and *NF-kB* gene expression (Figure 4, panels A and B). Moreover, the single constituents, as well as their combinations, MIX-1, MIX-2 and MIX-3, were able to decrease *TNF-α* gene expression (Figure 4, panel C). Interestingly, MIX-3 was more effective at decreasing the mRNA levels of all pro-inflammatory markers investigated (Figure 4), thus confirming the cardioprotective effects induced by the single extracts, as well as by their combinations.

Accordingly, Burja and collaborators (2019) showed that olive leaf extract was able to inhibit levels of pro-inflammatory cytokines, such as TNF-α, IL-1β, IL-6, and IL-8, in human arterial endothelial cells [58]. In addition, oleuropein, suggested to be the major component responsible for the anti-inflammatory effects of olive leaf extract, reduced COX-2 gene expression, as well as that of NF-kB signaling pathways also involved in the inflammation process [59]. Furthermore, water extracts of *S. baicalensis* were also found to suppress the production of several pro-inflammatory cytokines, including TNF-α, IL-6, and IL-1β, as well as COX-2 mRNA levels, in LPS-induced macrophages [60].

Interestingly, the combination of *S. baicalensis* and *Sophora japonica* L. was found to exert antioxidant and anti-hypertensive activities [61]. Furthermore, the combination of oleuropein, hydroxytyrosol, and oleocanthal induces cardioprotective, and anti-atherosclerotic activities in vivo in mice [62].

Our data align with recent pharmacological trends emphasizing the use of natural multi-component formulations to manage subclinical cardiac inflammation. The downregulation of pro-inflammatory mediators observed in our model provides a strong rationale for further investigating the potential use of our combinations as a complementary strategy in the prevention of inflammatory-based cardiovascular diseases.

On the basis of these results, we performed a final step in our experimental paradigm, with the aim to further study the possible cardioprotective effects of MIX-1, MIX-2 and MIX-3, with particular regard to their activities on the gene expression of cardiac biomarkers.

In addition to pro-inflammatory and pro-oxidant factors, cTnI and cTnT, which are highly expressed in cardiomyocytes, were strongly associated with heart failure and CVD death [63]. Furthermore, an association was reported between increased levels of BNP and a greater risk for adverse short- and long-term outcomes in heart failure. Moreover, BNP levels were found to be increased in perimyocarditis [64].

**Figure 5:**
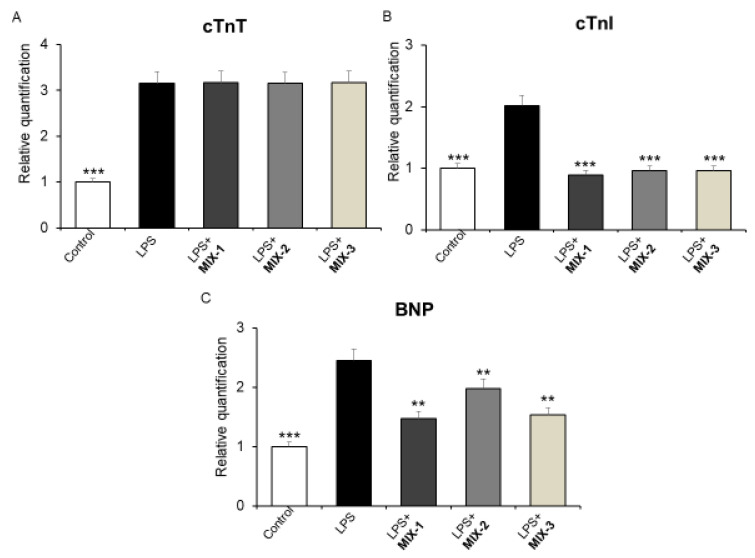
Effects of MIX-1, MIX-2 and MIX-3 on LPS-induced cardiac troponin T (*cTnT*) (**A**), cardiac troponin I (*cTnI*) (**B**), and natriuretic peptide (*BNP*) (**C**) gene expression (RQ, relative quantification), in mouse heart specimens (*n* = 30, 6 for each experimental group). Data are shown as the mean ± SEM. Statistical significance was calculated by one-way ANOVA followed by a Bonferroni post-test. ** *p* < 0.005; *** *p* < 0.01 vs. LPS.

In the present study, we showed that the combination inhibited the LPS-induced gene expression of *cTnI* and *BNP* in mouse heart specimens at all concentrations (Figure 5, panel B and C), without affecting *cTnT* gene expression.

Interestingly, cTnI is considered the preferred cardiac-specific biomarker for detecting cardiac injury, and increased cTnI was observed in myocardial injury [65]. Accordingly, herbal extracts were found to be effective at decreasing cTnI serum levels in diabetic rats; this could be related to their protective activities in diabetic cardiomyopathy [66]. Notably, pretreatment with oleuropein was able to counteract the increased serum levels of cTnI in rats with acute myocardial infarction [67], thus confirming its cardioprotective activity. While this does not directly indicate a reduction in *cTnI* gene expression, we can suggest that the suppression of LPS-induced cTnI mRNA levels following treatment with our combination could be related to its potential protective effects on the heart. Moreover, considering the experimental in vitro and ex vivo paradigms used, it was not possible to measure circulating levels of the same markers.

Our findings are also in agreement with those of Asghari and collaborators (2022), who reported that oleuropein could exert protective effects in contrasting fibrosis and reducing the gene expression of markers of myocardial hypertrophy, such as atrial natriuretic peptide and BNP, thus improving cardiac function in streptozotocin-induced diabetic rats [68]. The significant reduction in BNP and cTnI mRNA levels suggests that MIX-1, MIX-2 and MIX-3 could exert protective effects against the structural and functional myocardial stress induced by LPS.

### 3.4. Preventive Effects of MIX-1, MIX-2 and MIX-3 Against H_2_O_2_-Induced Intracellular ROS Production

Oxidative stress has been suggested to be involved in several CVDs, including heart failure, myocardial infarction, and ischemia/reperfusion damage [69].

In particular, ROSs play a pivotal role in the stimulation of endothelial dysfunction, vascular remodeling, and inflammation [70]. In agreement, increased cellular ROS production was shown to be involved in LDL oxidation [71], as well as in the pathogenesis and development of several CVDs such as atherosclerosis and heart failure [2,72]. Furthermore, higher cardiac troponin levels are strongly associated with cardiac muscle damage or stress. Moreover, H_2_O_2_ could contribute to this damage by enhancing oxidative stress, particularly in myocardial infarction or stress-induced cardiomyopathy [72].

**Figure 6:**
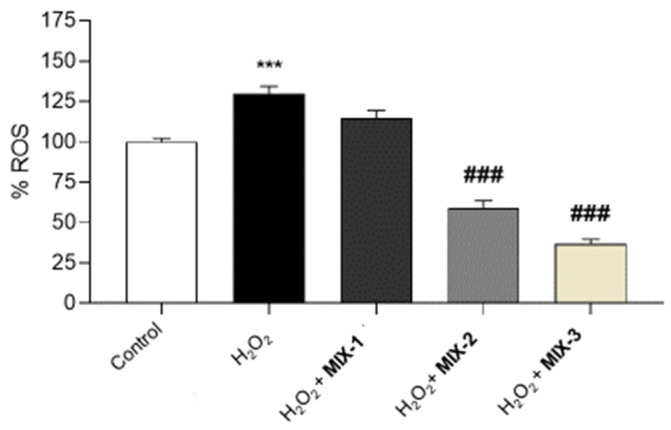
Evaluation of the preventive effects of MIX-1, MIX-2 and MIX-3 against H_2_O_2_-induced intracellular ROS production. The histograms show the levels of intracellular ROS (%) in H9c2 pre-treated with a control or MIX-1, MIX-2 and MIX-3, and then exposed to H_2_O_2._ All the experiments were performed in triplicate at minimum (*n* = 9). Data are displayed as the mean ± SEM. * indicates significant statistical differences vs. the control (*** *p* < 0.001), while # indicates statistical differences vs. the H_2_O_2_ treated-cells pre-incubated with the control (^###^ *p* < 0.001).

Our present findings showed that the treatment of H9c2 cells with H_2_O_2_ significantly increases intracellular ROS levels. However, pre-incubation with MIX-2 and MIX-3 significantly reduced ROS levels, suggesting preventive effects induced by the combination. The observed reduction is not significant at the lower concentration (MIX-1) (Figure 6).

Ultimately, the observed cardioprotective activities were not found to be associated with a single constituent but rather with various bioactive compounds. In this context, we can speculate that oleuropein from *Olea europaea* may act in combination with baicalin from *S. baicalensis* to inhibit pro-inflammatory and pro-oxidant signaling pathways. Moreover, the inclusion of policosanols may contribute to the stabilization of cellular membranes, thereby creating a multi-target therapeutic approach that addresses inflammatory process, oxidative stress, and structural damage simultaneously.

However, it is important to specify that our in vitro and ex vivo experimental models could not reflect, in vivo, the effects of the tested preparations on living organisms, due to limited absorption in the intestines and the metabolism of the preparation.

## 4. Conclusions

In conclusion, our results demonstrated that the combination MIX-3 exerts protective activities on the heart, as confirmed by the inhibitory effects on multiple markers involved in inflammatory processes and oxidative stress, including NF-kB, COX-2, TNF-α, iNOS, and CAT, as well as cardiac injury markers, such as cTnI and BNP, in mouse heart specimens treated with LPS. The combination may contribute to reduce ROS production and cardiac inflammation, as well as modulate key cardiac biomarkers that play a crucial role in CVD diagnosis and prognosis.

These findings suggest that this plant-based combination could be a promising candidate for the development of innovative functional foods and nutraceutical strategies aimed at supporting cardiovascular health and preventing oxidative stress-related cardiac dysfunction.

Further studies, including clinical trials, are needed to further confirm the activity of the combination as well as to evaluate its safety.

## Figures and Tables

**Table 1:** Characteristics of natural ingredients. * HPLC (high-performance liquid chromatography); GC (gas chromatography).

**Product Name:**	*Olea europaea*	*S. baicalensis*	Policosanols
**Botanical family**	Oleaceae	Lamiaceae	Poaceae
**Botanical name**	*Olea europaea* L.	*S. baicalensis* Georgi	*Saccharum officinarum* L.
**Origin**	Italy	China	China
**Part of plant used**	Leaf and Fruit	Root	Sugar cane juice
**Assay**	Oleuropein 15.0%	Baicalin 30.2%	Octacosanol 62.77% Hexacosanol 13.14% Tetracosanol 1.20% Dotriacotanol 1.0% Triacontanol 15.30% Nonacosanol 3.60% Heptacosanol 3.41%
Method Assay *	HPLC	HPLC	GC
**Description**	Powder	Powder	Powder
**Color**	Brown	Yellow-brown	White
**Particle size**	>80 mesh	≥90% through 80 Mesh	<80 mesh
**Extraction** **solvent**	Water	Ethanol/Water	Ethanol

## Data Availability

The original contributions presented in this study are included in the article/Supplementary Materials. Further inquiries can be directed to the corresponding author.

## Supplementary Materials

The following supporting information can be downloaded at https://www.mdpi.com/article/10.3390/foods15091500/s1.

## Abbreviations

The following abbreviations are used in this manuscript:

CVD	Cardiovascular diseases
BNP	B-type natriuretic peptide
cTnI	Cardiac troponin I
cTnT	Cardiac troponin T
ROS	Reactive oxygen species
LPS	Lipopolisaccharide
GPx	Glutathione peroxidase
CAT	Catalase
iNOS	Inducible nitric oxide synthase
COX-2	Cyclooxygenase-2
TNFα	Tumor necrosis factor α
NFkB	Nuclear factor-kB
IL	Interleukin
