# Supplementary material for: In Vitro and Ex Vivo Evaluation of a Multi-Target Combination of Plant Extracts and Policosanols: Effects in Mitigating Heart Inflammation and Oxidative Stress
Source: Foods. 2026 Apr 25;15(9):1500. doi: 10.3390/foods15091500 (PMC13164558; doi:10.3390/foods15091500)
Supplement: Supplementary file 1 [file foods-15-01500-s001.zip › foods-4241185-supplementary.pdf]

foods

Article

In Vitro and Ex Vivo Evaluation of a Multi-Target Combination of Plant Extracts and Policosanols: Effects in Mitigating Heart Inflammation and Oxidative Stress

Lucia Recinella [1,†], Giorgia Bray [2,†], Angelica Pia Centulio [1], Davide Ciaramellano [1,3], Annalisa Chiavaroli [1], Gianluca Genovesi [1], Giustino Orlando [1], Alessandra Acquaviva [1], Valentina Citi [2,4], Serena Veschi [1], Anna Piro [1], Alessandro Cama [1], Alma Martelli [2,4,5], Vincenzo Calderone [2,4,5], Luigi Brunetti [1,*] and Sheila Leone [1]

1 Department of Pharmacy, G. d'Annunzio University of Chieti-Pescara, 66013 Chieti, Italy; lucia.recinella@unich.it (L.R.); angelicapia.centulio@phd.unich.it (A.P.C.); d.ciaramellano@unidav.it (D.C.); annalisa.chiavaroli@unich.it (A.C.); gianluca.genovesi@phd.unich.it (G.G.); giustino.orlando@unich.it (G.O.); alessandra.acquaviva@unich.it (A.A.); veschi@unich.it (S.V.); anna.piro@unich.it (A.P.); alessandro.cama@unich.it (A.C.); sheila.leone@unich.it (S.L.)
2 Department of Pharmacy, University of Pisa, 56126 Pisa, Italy; giorgia.bray@phd.unipi.it (G.B.); valentina.citi@unipi.it (V.C.); alma.martelli@unipi.it (A.M.); vincenzo.calderone@unipi.it (V.C.)
3 Department of Human Sciences, Law, and Economics, Telematic University "Leonardo Da Vinci", UNIDAV, 66100 Torrevecchia Teatina, Italy
4 Interdepartmental Research Center "Nutrafood: Nutraceutica e Alimentazione per la Salute", University of Pisa, 56126 Pisa, Italy
5 CISUP (Centre for Instrumentation Sharing of Pisa University), 56126 Pisa, Italy
* Correspondence: luigi.brunetti@unich.it; Tel.: +39-0871-3554758
† These authors equally contributed to the manuscript.

1. Materials and Methods

1.1 The Characteristics of natural ingredients

Olea europaea (leaf and fruits (total oleuropein content = 15% p/p), *S. baicalensis* root (total baicalin content = 30.2 % p/p), and policosanols (total octacosanol content = 62.77%) extracts were supplied as dried powder by Difass International S.p.a. (Coriano, Rimini, Italy). *Olea europaea* and *S. baicalensis* extracts were dissolved in sterile phosphate buffer solution (PBS). Policosanols were dissolved in 30:70 (ehanol:water) solution. Then, for the experimental evaluations, the stock solution was dissolved in sterile PBS.

1.2. Toxicological and Pharmacological Studies

1.2.1. Cell Cultures

H9c2 cells (immortalized rat cardiomyoblasts, ATCC, Manassas, VA, USA) were cultured in Dulbecco's modified Eagle Medium with high glucose (DMEM-HG, Sigma-Aldrich, St. Louis, MO, USA) supplemented with 10% Fetal Bovine Serum (FBS), 1% penicillin-streptomycin solution containing 10,000 units of penicillin and 10 mg/ml of streptomycin (Sigma-Aldrich, St. Louis, MO, USA). Cells were incubated in T75 red cap tissue culture flasks, at 37°C in a humified atmosphere with 5% CO_2. Cells were used at approximately 80% confluency for each experiments.

1.2.2. Evaluation of Effects of *Olea europaea*, *S. baicalensis* and policosanols extracts on Cell Viability

 https://doi.org/10.3390/foods15091500

H9c2 cells were seeded in a 96-well cell culture plate at a density of 20 x 10^3 cells per well. After 24 hours (h), the culture medium was replaced, and the cells were treated with *Olea europaea* (5, 50 and 500 μg/ml), *Scutellaria baicalensis* (10, 100 and 1000 μg/ml), and policosanols (1.5, 15 and 150 μg/ml). After further 24 h, cell viability was assessed using Water Soluble Tetrazolium-1 (WST-1) colorimetric assay. WST-1 was added at a ratio of 1:10 and after 1 h of incubation at 37°C, absorbance at 450 nm was measured using spectrophotometer (Enspire, Perkin Elmer, USA). Cell viability was expressed as a percentage (%) versus control (culture medium).

1.2.3. Preventive Effects of *Olea europaea*, *S. baicalensis* and policosanols extracts, and their combination in an In Vitro Model of H_2O_2-Induced Oxidative Stress

H9c2 cells were seeded in a 96-well cell culture plate with a density of 20 x 10^3 cells per well. After 24 h, the culture medium was replaced, and the cells were treated with control or with the extracts tested individually as follows: *Olea europaea* (5, 50 and 500 μg/ml), *S. baicalensis* (10, 100 and 1000 μg/ml), and policosanols (1.5, 15 and 150 μg/ml). In addition, MIX-1 [*Olea europaea* (5 μg/ml) + *Scutellaria baicalensis* (10 μg/ml) + policosanols (1.5 μg/ml)], MIX-2 [*Olea europaea* (50 μg/ml) + *Scutellaria baicalensis* (100 μg/ml) + policosanols (15 μg/ml)] and MIX-3 [*Olea europaea* (500 μg/ml) + *Scutellaria baicalensis* (1000 μg/ml) + policosanols (150 μg/ml)] were also tested.

After 24 h, without removing the previous treatments, a freshly prepared hydrogen peroxide solution (H_2O_2) 100 μM was added for 2 h [1]. At the end of the treatment, the cell viability was assessed using the cell proliferation probe WST-1 (Roche, Basilea, Switzerland). WST-1 was added at a ratio of 1:10 and after 1 h of incubation at 37°C. Misuration of the absorbance at 450 nm was performed by a spectrophotometer (Enspire, Perkin Elmer, USA). Cell viability was expressed as a percentage (%) versus control (culture medium).

1.3. Ex vivo studies

1.3.1. Animals and Ethical Directives

Adult C57/BL6 male mice (3-month-old, weight 20–25 g) were housed in Plexiglas cages (2–4 animals per cage; 55 cm x 33 cm x 19 cm) and maintained under standard laboratory conditions (21 ± 2 °C; 55 ± 5% humidity) on a 14/10 h light/dark cycle, with ad libitum access to water and food. Housing conditions and experimentation procedures were strictly in agreement with the European Community ethical regulations (EU Directive no. 26/2014) on the care of animals for scientific research. In agreement with the recognized principles of "Replacement, Refinement and Reduction in Animals in Research", heart specimens were obtained as residual material from control-treated mice randomized in our previous experiments, approved by local ethical committee ('G. d'Annunzio' University, Chieti, Italy) and Italian Health Ministry (Project no. F4738.N.ZDZ).

1.3.2. Animal Protocol

After collection, isolated heart specimens were maintained in a humidified incubator with 5% CO_2 at 37 °C for 4 h (incubation period), in RPMI buffer with added bacterial LPS

(10 μg/mL), as previously described [2,3]. During the incubation period, the heart tissues were challenged with *Olea europaea* (5, 50 and 500 μg/ml), *Scutellaria baicalensis* (10, 100 and 1000 μg/ml), policosanols (1.5, 15 and 150 μg/ml), MIX-1 [*Olea europaea* (5 μg/ml) + *Scutellaria baicalensis* (10 μg/ml) + policosanols (1.5 μg/ml)]; MIX-2 [*Olea europaea* (50 μg/ml) + *Scutellaria baicalensis* (100 μg/ml) + policosanols (15 μg/ml)] and MIX-3 [*Olea europaea* (500 μg/ml) + *Scutellaria baicalensis* (1000 μg/ml) + Policosanols (150 μg/ml)] or control.

1.3.3. RNA Extraction

Total RNA was extracted from the colon specimens using TRI Reagent (Sigma–Aldrich, St. Louis, MO), according to the manufacturer's instructions. Briefly, each colon sample was homogenized in 1 ml of TRI Reagent. The homogenate was centrifuged at 12,000 × g for 10 min at 4 ◦C to remove the insoluble material. The supernatant, containing RNA and proteins, was added to 0.2 ml chloroform, then shaken vigorously and incubated on ice for 15 min. The next centrifugation at 12,000 × g for 15 min at 4 ◦C separated the mixture into three phases: a red organic phase, containing proteins, an interphase, containing DNA, and a colorless upper aqueous phase, containing RNA. The aqueous phase was removed and RNA was precipitated with 0.5 ml of isopropanol, stored for 30 min at −20 ◦C and pelleted by centrifugation at 12,000 × g for 10 min at 4 ◦C. The RNA pellet was washed with 1 ml of 75% ethanol, air dried and finally resuspended in RNase-free water. Contaminating DNA was removed using 2 units of RNase-free DNase-1 (DNA-free kit, Ambion, Austin, TX), according to the manufacturer's protocol. RNA purity and concentration were determined by spectrophotometry (BioPhotometer Eppendorf, Hamburg, Germany). In particular, RNA concentration was determined by spectrophotometer reading at 260 nm and its purity was assessed by the ratio at 260 and 280 nm readings. In order to evaluate the quality of extracted RNA, the samples were tested by electrophoresis through agarose gels and visualized by staining with ethidium bromide, under UV light.

1.3.4. Reverse transcription and real-time reverse transcription polymerase chain reaction (real-time RT PCR)

High capacity cDNA Reverse Transcription Kit (ThermoFischer Scientific, Waltman, Massachusetts, USA) was used to reverse transcribe RNA extracted from each colon specimen. In order to reverse transcribe 1 g of RNA in a reaction volume of 20 l, the reaction master mix was prepared according to the manufacturer's protocol. Reactions were incubated in a 2720 Thermal Cycler (ThermoFischer Scientific, Waltman, Massachusetts, USA) initially at 25 ◦C for 10 min, then at 37 ◦C for 120 min and finally at 85 ◦C for 5 s. Gene expression was determined by quantitative real-time PCR using TaqMan probe-based chemistry (ThermoFischer Scientific, Waltman, Massachusetts, USA). Reactions were performed in MicroAmp Fast Optic 96-well Reaction Plates (ThermoFischer Scientific, Waltman, Massachusetts, USA) on an ABI PRISM 7900 HT Fast Real-Time PCR System (ThermoFischer Scientific, Waltman, Massachusetts, USA), in "fast" operational mode. PCR primers and TaqMan probes, including β-actin used as the housekeeping gene, were purchased from Thermo Fisher Scientific Inc. (Assays-on-

Demand Gene Expression Products, Mm00656767_g1 for GPX gene, Mm00437992_m1 for CAT gene, Mm00440502_m1 for iNOS gene, Mm00478374_m1 for COX-2 gene, Mm00476361_m1 for NF-kB gene, Mm00443258_m1 for TNF-α gene, Mm00437164_m1 for cTnI, Mm01290256_m1 for cTnT, Mm01255770_g1 for BNP, Mm00607939_s1 for β-actin gene). β-actin was used as the housekeeping gene. Each amplification reaction was performed with 10 l of TaqMan Fast Universal PCR Master Mix (2×), No AmpErase UNG (ThermoFischer Scientific, Waltman, Massachusetts, USA), 1 µl of primer probe mixture, 1 µl of cDNA and 8 µl of nuclease-free water, according to the manufacturer's protocol. Real-time PCR was carried out in triplicate for each cDNA sample in relation to each of the investigated genes. No-template control, one for each Assay-on-Demand, was used to check for contamination. A reverse transcriptase minus control was included for β-actin gene Assay-on-Demand. The thermal cycling conditions were: 95 °C for 20 s, followed by 40 cycles of amplification at 95 °C for 1 s and 60 °C for 20 s. Sequence Detection System (SDS) software – version 2.3 – (ThermoFischer Scientific, Waltman, Massachusetts, USA) elaborated gene expression data. The comparative 2−ΔΔCt method was used to quantify the relative abundance of mRNA and then determine the relative changes in individual gene expression (relative quantification, RQ). This method uses a calibrator sample to enable a comparison of gene expression levels in different samples. The values obtained indicate the changes in gene expression in the sample of interest by comparison with the calibrator sample, after normalization to the housekeeping gene.

1.3.4. Antioxidant effects of *Olea europaea, Scutellaria baicalensis* and policosanols extracts combination in an in vitro model of H_2O_2-induced oxidative stress

H9c2 cells were seeded at 30 x 10^3 cells/well in 96-well black plate coated with a 1% aqueous solution of gelatin (Sigma-Aldrich, St. Louis, MO, USA). After 24 h cells were treated with MIX-1, MIX-2 and MIX-3.

After 24 h, without removing the previous treatments, a freshly prepared H_2O_2 solution 100 µM was added for 2 h. At the end of the treatment, the culture medium was removed, and reactive oxygen species (ROS) were detected using the fluorescence probe dihydroethidium (DHE) (Sigma-Aldrich, St. Louis, MO, USA). DHE was prepared at the concentration of 10 µM in Hanks' Balanced Salt solution (HBSS) (Sigma-Aldrich, St. Louis, MO, USA). After 30 minutes of incubation at 37°C, the fluorescence at 500 nm excitation 580 nm emission was detected using a microplate reader (Enspire, Perkin Elmer, USA). The results were expressed as a percentage (%) versus control (culture medium).

1.4. Statistical analysis

Statistical analyses were performed using GraphPad Prism 8.0.2. Data are expressed as Mean ± standard error (SEM). Three different experiments were performed, each with three replicates (n = 9). Data were analysed using one-way analysis of variance (ANOVA), followed by Bonferroni test. Statistically significant differences were defined as $p<0.05$.

The number of animals randomized for each experimental group was calculated on the basis of the "Resource Equation" $N = (E + T)/T$ ($10 \le E \le 20$) [4].

https://doi.org/10.3390/xxxxx

References

1. Panera, N.; Gnani, D.; Piermarini, E.; Petrini, S.; Bertini, E.; Nobili, V.; Pastore, A.; Piemonte, F.; Alis.i A. High concentrations of H2O2 trigger hypertrophic cascade and phosphatase and tensin homologue (PTEN) glutathionylation in H9c2 cardiomyocytes. *Exp Mol Pathol.* **2016,** *10,* 199-206.
2. Recinella, L.; Chiavaroli, A.; Masciulli, F.; Fraschetti, C.; Filippi, A.; Cesa, S.; Cairone, F.; Gorica, E.; De Leo, M.; Braca, A.; et al. Protective Effects Induced by a Hydroalcoholic Allium Sativum Extract in Isolated Mouse Heart. *Nutrients* **2021**, *13*, 2332.
3. Recinella, L.; Libero, M.L.; Citi, V.; Chiavaroli, A.; Martelli, A.; Foligni, R.; Mannozzi, C.; Acquaviva, A.; Di Simone, S.C.; Calderone, V.; et al. Anti-Inflammatory and Vasorelaxant Effects Induced by an Aqueous Aged Black Garlic Extract Supplemented with Vitamins D, C, and B12 on Cardiovascular System. *Foods* **2023**, *12*, 1558.
4. Charan, J.C.; Kantharia, N.D.K. How to Calculate Sample Size in Animal Studies? *J Pharmacol Pharmacother* **2013**, *4*, 303–306.

Disclaimer/Publisher's Note: The statements, opinions and data contained in all publications are solely those of the individual author(s) and contributor(s) and not of MDPI and/or the editor(s). MDPI and/or the editor(s) disclaim responsibility for any injury to people or property resulting from any ideas, methods, instructions or products referred to in the content.
